# Predictive Value of a New Muscle Parameter in Patients with Resectable Gastric Cancer: A Pooled Analysis of Three Prospective Trials

**DOI:** 10.1245/s10434-024-14913-w

**Published:** 2024-01-25

**Authors:** Qing Zhong, Jiao-Bao Huang, Jun Lu, Li-Wei Xue, Guang-Tan Lin, Jian-Wei Xie, Jian-Xian Lin, Chao-Hui Zheng, Chang-Ming Huang, Ping Li

**Affiliations:** 1 Department of Gastric Surgery, Fujian Medical University Union Hospital, Fuzhou, Fujian China; 2 Department of Gastrointestinal Surgery, The Second Affiliated Hospital of Nanchang University, Nanchang, China; 3 Department of Radiology, Fujian Medical University Union Hospital, Fuzhou, China

## Abstract

**Background:**

Sarcopenia is closely associated with gastric cancer (GC) prognosis. However, its exact definition remains controversial.

**Methods:**

This study included computed tomography images and clinical data of patients from three prospective studies. The skeletal muscle index (SMI) and skeletal muscle radiation attenuation (SMRA) were analyzed, and a new muscle parameter, skeletal muscle gauge (SMG), was obtained by multiplying the two parameters. The values of the three indices for predicting the prognosis of patients with GC were compared.

**Results:**

The study included 717 patients. The findings showed median values of 42 cm^2^/m^2^ (range, 36.8–48.2 cm^2^/m^2^) for SMI, 45 HU (range, 41–49 HU) for SMRA, and 1842 (range, 1454–2260) for SMG. Postoperatively, 111 patients (15.5%) experienced complications. The 3-year overall survival (OS), disease-free survival (DFS), and recurrence-free survival (RFS) were 74.3%, 68.2%, and 70%, respectively. Univariate logistic analysis showed that postoperative complications were associated with SMI (odds ratio [OR] 0.94; 95% confidence interval [CI] 0.92–0.96), SMRA (OR, 0.87; 95% CI 0.84–0.90), and SMG (OR 0.99; 95% CI 0.98–0.99). After a two-step multivariate analysis, only SMG (OR 0.98, 95% CI 0.97–0.99) was an independent protective factor of postoperative complications. Multivariate analysis showed that SMG also was an independent protective factor of OS, DFS, and RFS. The patients were divided into low-SMG (L-SMG) group and high-SMG (H-SMG) groups. Chemotherapy benefit analysis of the patients with stage II low SMG showed that the OS, DFS, and RFS of the chemotherapy group were significantly better than those of the non-chemotherapy group (*p* < 0.05).

**Conclusion:**

The prospective large sample data showed that the new muscle parameter, SMG, can effectively predict the short-term outcome and long-term prognosis of patients with resectable gastric cancer. As a new muscle parameter index, SMG is worthy of further study.

**Supplementary Information:**

The online version contains supplementary material available at 10.1245/s10434-024-14913-w.

Gastric cancer currently is ranked fourth in terms of morbidity rate and fifth in terms of mortality rate worldwide.^1,2^ Sarcopenia is a common complication among patients with gastric cancer and often presents with weakness and malnutrition.^3,4^ Currently, the diagnosis of sarcopenia is primarily based solely on the specific cutoff point value of the skeletal muscle index (SMI). However, this is the most optimal approach for evaluation? Clearly, there is still ongoing controversy. First, age is a well-known factor that affects muscle quality, yet the conventional definition of sarcopenia fails to consider age as a factor.^5^ Second, the use of different cutoff points in various studies hinders study reproducibility and further exploration. Most importantly, SMI can represent only the quantity of skeletal muscle, but the skeletal muscle radiation attenuation (SMRA), which represents the quality of skeletal muscle, has not been fully considered.^6,7^

Skeletal muscle radiation attenuation represents the amount of radiation (expressed in Hounsfield units [HU]) absorbed by the tissue during computed tomography (CT) scanning. A lower HU value indicates a higher triglyceride concentration and worse muscle mass.^8^ Schneider et al.^9^ found that SMRA is more accurate than SMI in predicting short-term outcomes of colon cancer. However, whether the patients with gastric cancer still can maintain the advantage needs to be further explored. In addition, there are still some limitations to simply replacing SMRA with SMI. For example, SMRA can represent only the quality of skeletal muscles without considering their quantity.

Recent studies have shown that the muscle parameter skeletal muscle gauge (SMG) obtained by multiplying SMI and SMRA is superior to SMI or SMRA alone in predicting adverse outcomes for some tumor patients.^10^ The SMG provides a comprehensive measure of the quality and quantity of individual skeletal muscles, similar to how the value of a diamond depends on its weight and purity. But the SMG is rarely reported in gastric cancer.

There is an urgent need for high-quality evidence-based medicine data to confirm the value of SMG for patients with gastric cancer. Therefore, we performed a pooled analysis of three previously reported prospective clinical cohorts of patients (CLASS-04, FUGES-001, and FUGES-002) who underwent laparoscopic gastric cancer surgery. By re-evaluating muscle parameters using CT scans and clinical data, we compared the impact of the three muscle parameters on the short- and long-term outcomes of patients with resectable gastric cancer.

## Methods

### Study Population and Data Collection

This study examined 1225 patients who participated in three prospective clinical studies conducted at the Department of Gastric Cancer, affiliated with Fujian Medical University Union Hospital, between January 2015 and December 2018. All procedures followed the ethical standards of the responsible committee on human experimentation (institutional and national) and the Helsinki Declaration of 1964 and later versions. Informed consent or a substitute for it was obtained from all patients for their inclusion in the study.

The CLASS-04 study (NCT02845986) enrolled 251 patients between 14 September 2016, and 12 October 2017. The study aimed to assess the safety and feasibility of laparoscopic spleen-preserving lymph node dissection performed by the Chinese Laparoscopic Gastrointestinal Surgery Study Group (CLASS) in patients with locally advanced gastric cancer.^11^

The FUGES-001 study (NCT02845986) recruited 438 patients from 1 January 2015, to 1 April 2016. Its objective was to compare the safety and efficacy of three-dimensional (3D) and two dimensional (2D) laparoscopic gastrectomy in gastric cancer.^12^

The FUGES-002 study evaluated the surgical outcomes of laparoscopic spleen-preserving no. 10 lymphadenectomy and enrolled 536 patients between 5 January 2015, and 10 December 2018.^13^

All three studies were approved by the local ethics committee. Surgical techniques, perioperative management, determination of study end points, and results of these studies have been previously reported. All three prospective trials had similar inclusion and exclusion criteria (eTables 1, 2, and 3).

Except for the tumor location and clinical T stage specified in each regimen, patients requiring neoadjuvant therapy were not included in our study. Patients who had undergone radical gastrectomy were eligible to participate in this combined analysis. The exclusion criteria ruled out patients who withdrew their consent, patients proven unable to complete R0 resection during operation, lack of access to CT imaging data, the 3D laparoscopy group in the FUGES-01 study, and patients with a history of lumbar surgery.

**Fig. 1:**
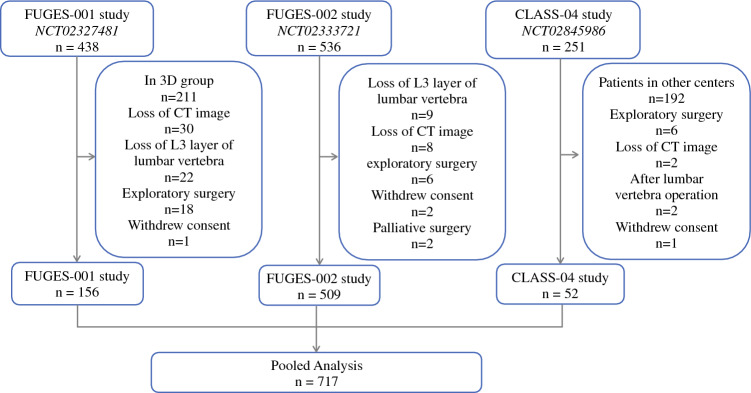
Study flowchart.

Of the 1225 patients, 717 met the criteria for inclusion in this pooled analysis (Fig. 1). Heterogeneity among the three studies was minimized using similar laparoscopic procedures. All the resected specimens were pathologically evaluated according to a standardized method.^14^ All the patients underwent the same perioperative management and follow-up plans.

### Skeletal Muscle Parameters

A single CT image at the level of the third lumbar vertebra (L3) was chosen for muscle quantification due to its close association with the overall body volume.^15^ Two researchers reached a consensus on the accurate acquisition of L3 images and muscle segmentation. In case of any doubts during the muscle segmentation process, they sought guidance from a third expert.

The CT images used for analysis did not include any patient-specific information. The sliceOmatic 5.0 version software (TomoVision, Toronto, ON, Canada) was used for the analysis of these images.^16^ According to the standard HU range, the cross-sectional area of the skeletal muscle (SMA, cm^2^) was converted to SMI after division of the measured SMA by the square meter of the height,^17^ and the muscle quality was reflected by SMRA (calculated in HU). Body mass index (BMI) was calculated by dividing weight by height squared (kg/m^2^). In the analysis, BMI was divided into two groups: a low-BMI (< 25 kg/m^2^) group and a high-BMI (≥25 kg/m^2^) group.^18^ The SMG was derived by multiplying the SMI and the SMRA. Based on the overall survival (OS) rate, the SMG was divided into two groups: a high-SMG group (H-SMG) and a low-SMG group (L-SMG). The cutoff point for differentiating between these groups was determined using X-tile analysis, taking into consideration the sex of the individuals.

### Surgical Quality Control and Follow-up Evaluation

All the patients underwent surgery performed by two experienced surgeons (C-M. H. and C-H. Z) who had passed the learning curve and completed more than 50 laparoscopic radical surgeries for gastric cancer. The surgical procedures performed followed the Japanese guidelines for the treatment of gastric cancer, which involved laparoscopic radical gastrectomy and D2 lymphadenectomy.^19^ Morbidity and mortality were evaluated within 30 days after surgery. Postoperative complications were graded according to the Clavien-Dindo classification.^20^

Each patient underwent at least 36 months of follow-up evaluation. The patients were followed up every 3 months in the first 2 years and every 6 months in the following 3 years. Most routine follow-up appointments included physical examinations, laboratory examinations (including CA19-9, CA72-4, and CEA measurements), chest radiography, abdominal and pelvic ultrasound or computed tomography, and annual endoscopy.

Overall survival was defined as death from randomized to death from any cause. Disease-free survival rate (DFS) was defined as the time from randomized to the emergence of evidence of disease recurrence or the time at which the subject died for any reason.^21^ Recurrence-free survival rate (RFS) was defined as the interval between the first treatment and tumor recurrence.^21^

### Nutritional Support

According to the recommendations of the European Society for Clinical Nutrition and Metabolism, all patients used Nutritional Risk Screening 2002 (NRS2002) to receive nutritional risk screening^22^ and developed personalized nutritional support therapy. Patients with an NRS score of 3 or higher were routinely given oral nutritional supplements. Patients who could not meet their energy needs through oral feeding were provided with enteral tube feeding, parenteral nutrition, or both.^23^ In addition, all patients underwent nutritional assessment every 2 weeks to adjust their nutritional support treatment until 1 week before surgery.

### Statistical Analysis

The data are described as the absolute number and percentage of normally distributed variables, as the mean and standard deviation (SD), or as the median and interquartile range (IQR). The classified variables were tested by the chi-square test or Fisher’s exact test, and the continuous variables were compared by *t* test. Delayed chemotherapy was defined as occurring longer than 8 weeks after surgery.^24^ Pearson’s correlation coefficient was used to analyze the correlation between the parameters and age. Two-step logistic regression and Cox regression models were used for multivariate analysis. The receiver operating characteristic (ROC) area under the curve (AUC), the C-index, the Akaike information criterion (AIC), and the net reclassification index (NRI) were used to compare the predictive performance of the skeletal muscle parameters for short-term efficacy and long-term survival in the three groups.

Decision curve analysis (DCA) can intuitively judge the threshold probability of the prediction model to test its clinical value. The best SMG cutoff value was obtained using X-tile (developed at Yale University as a bio-informatics tool for biomarker assessment and outcome optimization^25^; the working principle is to distinguish the final population subsets and the associated Kaplan-Meier curve through the log-rank test).

A *p* value lower than 0.05 was considered statistically significant. For statistical analysis, SPSS software (version 22.0, Stanford, CA, USA) and R version 3.6.0 (R Foundation for Statistical Computing, Auckland, New Zealand) were used.

## Results

### Baseline Clinicopathologic Data

The study analyzed 717 patients, including 528 male patients (73.6%) and 189 female patients (26.4%) with a median age of 62 years (interquartile range [IQR], 55–67 years), an Eastern Cooperative Oncology Group (ECOG) score of 1 or higher in 313 cases (43.7%), a BMI of 25 kg/m^2^ or higher in 136 cases (19%), and a tumor size of 5 cm or larger in 263 cases (36.3%). The intraoperative blood loss was 40 mL (IQR, 20–72 mL), and the operation time was 180 min (IQR, 155–208 min). The median hospital stay was 9 days (IQR, 8–12 days).

The postoperative pathologic staging included 108 (15.1%) patients with stage I disease, 182 (25.4%) patients with stage II disease, 427 (59.5%) patients with stage III disease. The 717 patients included 467 (65.1%) patients with chemotherapy and 250 (34.9%) patients without chemotherapy. The median time to the start of chemotherapy after operation was 4.9 weeks (range, 3.5–10 weeks). The findings showed median values of 42 cm^2^/m^2^ (IQR, 36.8–48.2 cm^2^/m^2^) for SMI, 45 HU (IQR, 41–49 HU) for SMRA, and 1842 (IQR, 1454–2260) for SMG (Table 1).

**Table 1:** General characteristics of patients

Characteristics	Total (*n* = 717) *n* (%)
Gender	
Male	528 (73.6)
Female	189 (26.4)
Age: years (IQR)	62 (55–67)
ECOG	
0	404 (56.3)
≥ 1	313 (43.7)
BMI (kg/m^2^)	
< 25	581 (81.0)
≥ 25	136 (19.0)
Tumor location	
Upper	443 (61.7)
Middle	196 (27.3)
Lower	78 (10.8)
Tumor size (cm)	
< 4	324 (45.2)
≥ 4	393 (54.8)
Intraoperative bleeding volume: ml (IQR)	40 (20–72)
Operation time: min (IQR)	180 (155–208)
pTNM stage	
I	108 (15.1)
II	182 (25.4)
III	427 (59.5)
Postoperative complication	
Yes	111 (15.5)
No	606 (84.5)
Clavien-Dindo classification	
I	2 (1.8)
II	72 (64.8)
III	26 (23.4)
IV	9 (8.1)
V	2 (1.8)
Adjuvant chemotherapy	
Yes	467 (65.1)
No	250 (34.9)
Hospitalization time: days (range)	9 (8–12)
Postoperative chemotherapy time: weeks (range)	4.9 (3.5–10.0)
Skeletal muscle index: cm^2^/m^2^ (IQR)	42 (36.8–48.2)
Skeletal muscle radiation attenuation: HU (IQR)	45 (41–49)
Skeletal muscle gauge (IQR)	1842 (1454–2260)

IQR, interquartile range; ECOG, Eastern Cooperative Oncology Group; BMI, body mass index; pTNM, pathologic tumor-node-metastasis

### Correlation Analysis Between Skeletal Muscle Parameters and Baseline Data

eFigure 1 shows representative L3 plane CT image segmentation with the patient’s baseline state, and eFigure 1A is the representative segmentation of normal SMI (42 cm^2^/m^2^) and SMRA (45 HU). eFigure 1B shows representative segmentation of low SMI (SMI, 27.4 cm^2^/m^2^; SMRA, 43.2 HU), and eFigure1 C shows the representative segmentation of low SMRA (SMI, 37.1 cm^2^/m^2^; SMRA, 28.7 HU).

Correlation analysis showed that age was weakly correlated negatively with SMRA and SMG (SMRA: Pearson’s *r,* − 0.200, *p* < 0.001; SMG: Pearson’s *r,* − 0.114, *p* < 0.001), whereas BMI and SMI did not correlate with age (BMI: Pearson’s *r,* 0.083, *p* = 0.065; SMI: Pearson’s *r,* − 0.330, *p* = 0.371).

In addition, we analyzed the relationship between SMG and BMI comorbidity and pathologic stages. The findings showed that the higher the BMI (< 25 vs ≥ 25), the higher the SMG (1799 vs 2004) (*p* < 0.001), the higher the pathologic stage (stage III vs. II), and the lower the SMG (1743 vs. 1958 vs 2142) (*p* < 0.001), but there was no correlation between SMG and comorbidity (comorbidity vs non-comorbidity: 1790 vs 1853; *p* = 0.163; eFigure 2).

### SMG Performance in Predicting Postoperative Complications Is Better Than That of SMI and SMRA

Among the total population, postoperative complications occurred in 111 cases (15.5%) and major complications (CD grade ≥ 3) in 37 cases (5.1%). The most common complications were pneumonia (71 patients, 9.9%), abdominal infection (23 patients, 3.2%), and anastomotic leakage (15 patients, 2.0%) (eTable 4). Univariate logistic regression analysis showed that age of 60 years or older, ECOG of 1 or higher, BMI of 25 kg/m^2^ or higher, and SMI, SMRA, and SMG were associated with postoperative complications (eTable 5).

In the two-step multivariate analysis, the independent protective factors of postoperative complications were only SMG (OR, 0.98; 95% CI 0.97–0.99), ECOG of 1 or higher (OR, 1.61; 95% CI 1.02–2.54), and age of 60 years or older (OR, 1.68; 95% CI 1.18–2.58; *p* = 0.035) (Table 2). By comparing the predictive performance of the three muscle parameters for postoperative complications using the ROC curve, we found SMG > SMRA > SMI (AUC value: SMG, 0.778; SMRA, 0.740; SMI, 0.651; eFig. 3A). The AIC analysis showed that SMG had better goodness of fit in predicting postoperative complications than SMI or SMRA (477.4 vs. 557.8 vs. 515.1; *p* < 0.001).

**Table 2:** Two-step multivariate analysis of postoperative complications

	Postoperative complications			
	95% CI			
	OR	Lower	Upper	*p* Value
Step 1^a^				
Age ≥ 60 years	1.73	1.11	2.68	**0.014**
ECOG ≥1	1.64	1.03	2.61	**0.037**
BMI ≥25 kg/m^2^	2.84	1.65	4.87	**0.041**
Operation time	1.01	0.99	1.01	0.072
SMI	0.94	0.92	0.96	**0.001**
Step 1^b^				
Age ≥ 60 years	1.86	1.21	2.45	**0.021**
ECOG ≥1	1.55	1.12	2.43	**0.029**
BMI ≥25 kg/m^2^	2.72	1.81	5.12	**0.045**
Operation time	1.13	0.99	1.02	0.081
SMRA	0.87	0.84	0.90	**0.001**
Step 1^c^				
Age ≥60 years	1.81	1.32	2.42	**0.021**
ECOG ≥1	1.53	1.12	2.54	**0.028**
BMI ≥25 kg/m^2^	2.68	1.82	4.66	**0.043**
Operation time	1.03	0.99	1.04	0.075
SMG	0.99	0.98	0.99	**0.001**
Step 2^d^				
Age ≥ 60 years	1.68	1.18	2.58	**0.035**
ECOG ≥1	1.61	1.02	2.54	**0.043**
SMG	0.98	0.97	0.99	**0.001**

Bold values indicate that the *P* value is statistically significant

CI, confidence interval; ECOG, Eastern Cooperative Oncology Group; BMI, body mass index; SMRA, skeletal muscle radiation attenuation; SMG, skeletal muscle gauge

^a^All significant factors in the univariate analysis were used for analysis except SMRA and SMG.

^b^All significant factors in the univariate analysis were used for analysis except SMI and G.

^c^All significant factors in the univariate analysis were used for analysis except SMI and SMRA.

^d^All significant factors in the univariate analysis were used for analysis including SMI, SMRA, and SMG.

Using the NRI to judge the predictive performance of SMG for postoperative complications, the predictive ability of SMG was found to be 72.9% higher than that of SMI and 65.3% higher than that of SMRA (Table 4). We also used a DCA to intuitively evaluate and compare the clinical applicability of the three groups of muscle parameters. The results showed that compared with SMI and SMRA under the same probability threshold, SMG can achieve a better net benefit for the prediction of postoperative complications (eFig. 3B).

### SMG is Superior to SMI and SMRA in Predicting Long-Term Outcomes

**Fig. 2:**
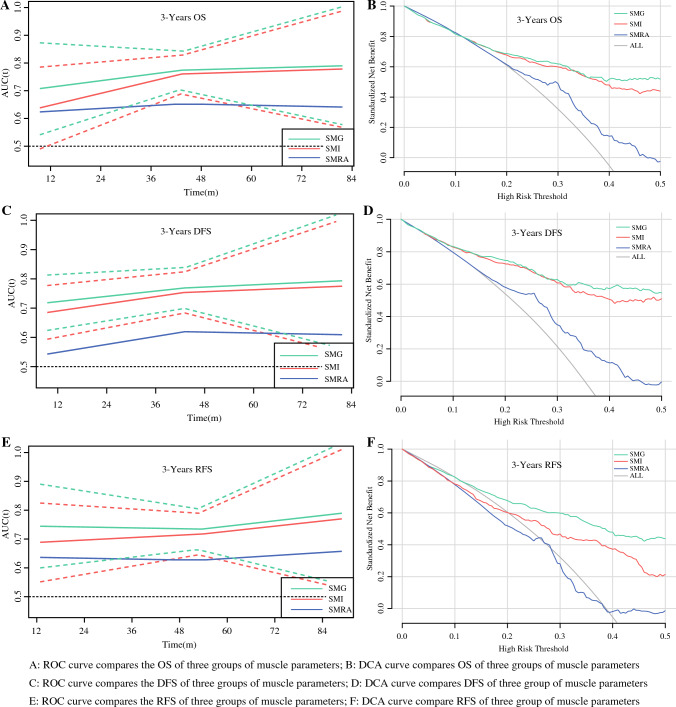
Time-dependent ROC and DCA curve of different skeletal muscle parameters on overall survival, disease-free survival, and recurrence-free survival. ROC, receiver operating characteristic; DCA, decision curve analysis

The median follow-up time was 53 months (IQR, 34–73 months). The findings showed a 3-year OS of 74.3%, a 3-year DFS of 68.2%, and a 3-year RFS of 70.0%. Univariate Cox regression analysis showed that a pTNM of II or higher, chemotherapy, a tumor size of 4 cm or larger, and SMI, SMRA, and SMG were associated with OS, DFS, and RFS (eTable 6). Two-step multivariate Cox regression analysis showed that pTNM, chemotherapy, and SMG were independent factors influencing OS, DFS, and RFS (Table 3). Compared using the time-dependent ROC curve, SMG was better than SMI and SMRA in predicting OS, DFS, and RFS among the three groups of muscle parameters (C-index: OS [SMI, 0.743; SMRA, 0.610; SMG, 0.761], DFS [SMI, 0.720; SMRA, 0.598; SMG, 0.728], RFS [SMI, 0.718; SMRA, 0.622; SMG, 0.755]; Fig. 2A, C, and E).

**Table 3:** Two-step multivariate analysis of overall survival, disease-free survival, and recurrence-free survival

	Overall survival				Disease-free survival				Recurrence-free survival			
	(95% CI)				(95% CI)				(95% CI)			
	HR	Lower	Upper	*p* Value	HR	Lower	Upper	*p* Value	HR	Lower	Upper	*p* Value
Step 1^a^												
Tumor size (≥ 4cm)	2.31	1.77	3.42	**0.001**	2.23	1.76	2.93	**0.001**	1.23	0.92	1.64	0.167
pTNM (stage II)	3.41	1.53	8.65	**0.011**	4.20	1.64	10.75	**0.003**	3.56	1.82	9.34	**0.003**
pTNM (stage III)	13.62	7.67	36.82	**0.001**	18.01	7.43	43.69	**0.001**	16.25	8.56	38.55	**0.001**
Adjuvant chemotherapy (No)	1.64	1.23	1.72	**0.005**	1.28	1.01	1.63	**0.043**	1.67	1.23	2.19	**0.001**
SMI	0.93	0.89	0.96	**0.001**	0.92	0.91	0.93	**0.001**	0.94	0.93	0.96	**0.001**
Step 1^b^												
Tumor size (≥ 4cm)	3.04	1.94	4.12	**0.001**	2.45	1.64	2.86	**0.001**	1.21	0.91	1.63	0.199
pTNM (stage II)	3.56	1.62	6.72	**0.025**	5.32	2.52	9.69	**0.004**	4.51	2.48	8.55	**0.008**
pTNM (stage III)	16.83	7.43	35.62	**0.001**	17.65	8.52	40.38	**0.001**	14.25	9.32	39.34	**0.001**
Adjuvant chemotherapy (No)	1.51	1.22	1.73	**0.007**	1.19	1.03	1.61	**0.034**	1.79	1.34	2.33	**0.001**
SMRA	0.92	0.90	0.96	**0.001**	0.96	0.95	0.97	**0.001**	0.97	0.95	0.98	**0.001**
Step 1^c^												
Tumor size (≥ 4cm)	2.21	1.62	3.45	**0.001**	2.31	1.82	2.82	**0.001**	1.16	0.87	1.56	0.318
pTNM (stage II)	2.89	1.43	7.56	**0.018**	4.42	1.72	10.61	**0.003**	3.81	1.61	9.53	**0.003**
pTNM (stage III)	17.85	8.21	34.21	**0.001**	16.42	8.51	38.71	**0.001**	15.82	9.45	36.62	**0.001**
Adjuvant chemotherapy (No)	1.34	1.20	1.51	**0.009**	1.46	1.11	1.51	**0.045**	1.72	1.32	2.23	**0.001**
SMG	0.99	0.97	0.99	**0.001**	0.99	0.97	0.99	**0.001**	0.99	0.98	0.99	**0.001**
Step 2^d^												
pTNM (stage II)	3.21	1.23	8.31	**0.017**	5.05	1.96	12.99	**0.001**	4.43	2.04	10.81	**0.001**
pTNM (stage III)	10.94	4.48	26.7	**0.001**	17.19	7.03	42.02	**0.001**	16.19	8.53	40.12	**0.001**
Adjuvant chemotherapy (No)	1.48	1.15	1.90	**0.002**	1.79	1.40	2.28	**0.001**	1.72	1.32	2.23	**0.001**
SMG	0.99	0.98	0.99	**0.001**	0.99	0.97	0.99	**0.001**	0.99	0.97	0.99	**0.001**

Bold values indicate that the *P* value is statistically significant

CI, confidence interval; HR, hazard ratio; pTNM, pathologic tumor-node-metastasis; SMI, skeletal muscle index; SMG, skeletal muscle gauge; SMRA, skeletal muscle radiation attenuation

^a^All significant factors in the univariate analysis were used for analysis except for SMRA and SMG

^b^All significant factors in the univariate analysis were used for analysis exceptfor SMI and SMG

^c^All significant factors in the univariate analysis were used for analysis except for SMI and SMRA

^d^All significant factors in the univariate analysis were used for analysis including SMI, SMRA and SMG;

According to AIC analysis, SMG had better goodness of fit than SMI or SMRA (OS [2959.3 vs. 2973.4 vs. 3165.4; *p* < 0.001], DFS [3300.1 vs 3321.4 vs. 3478.2; *p* < 0.001], RFS [SMI, 3310.5; SMRA, 3420.5; SMG, 3260.2]). The comparison of the NRI indices showed that the predictive ability of SMG for OS was 56.3% higher than that of SMI and 69.5% higher than that of SMRA; that for DFS was 53.3% higher than that of SMI and 65.4% higher than that of SMRA; and that for RFS was 51.5% higher than that of SMI and 62.1% higher than that of SMRA (Table 4). The results of the DCA showed that under the same probability threshold, compared with SMI and SMRA, SMG can achieve a better net income in the prediction of OS, DFS, and RFS (Fig. 2B, D, and F).

**Table 4:** Comparison of prognostic performance of muscle parameters in three groups

Variable	SMI	SMRA	SMG	*p* Value
Postoperative complications				
AUC^a^	0.651	0.740	0.778	**< 0.001**
AIC^b^	557.8	515.1	477.4	**< 0.001**
NRI (95% CI)^c^ SMI vs SMG			72.9% (53.6–91.8%)	**< 0.001**
NRI (95% CI)^c^ SMRA vs SMG			65.3% (26.6–73.7%)	**< 0.001**
Overall survival				
Harrell’s C index^a^	0.743	0.610	0.761	**< 0.001**
AIC	2973.4	3165.4	2959.3	**< 0.001**
NRI (95% CI) SMI vs SMG			56.3% (38.9–72.4%)	**< 0.001**
NRI (95% CI) SMRA vs SMG			69.5% (46.6–78.2%)	**< 0.001**
Disease-free survival				
Harrell’s C index	0.720	0.598	0.728	**< 0.001**
AIC	3321.4	3478.2	3300.1	**< 0.001**
NRI (95% CI) SMI vs SMG			53.3% (40.8–68.2%)	**< 0.001**
NRI (95% CI) SMRA vs SMG			65.4% (39.5–79.6%)	**< 0.001**
Recurrence-free survival				
Harrell’s C index	0.718	0.622	0.755	**< 0.001**
AIC	3310.5	3420.5	3260.2	**< 0.001**
NRI (95% CI) SMI vs SMG			51.5% (43.5–64.5%)	**< 0.001**
NRI (95% CI) SMRA vs SMG			62.1% (38.6.5–75.4%)	**< 0.001**

Bold values indicate that the *P* value is statistically significant

SMI, skeletal muscle index; SMRA, skeletal muscle radiation attenuation; SMG, skeletal muscle gauge AUC, area under the curve; AIC, Akaike information criterion; CI, Confidence interval; NRI, net reclassification index

^a^A higher AUC and Harrell’s C-index indicates higher discriminative ability.

^b^Smaller AIC values indicate better optimistic prognostic stratification.

^c^NRI quantifies the performance improvement of the new predictor.

### Comparison of Clinical Data and Benefit Analysis of Chemotherapy Between the Two Groups

The cutoff point value of the SMG through the X-tile was 1713 for the male and 1318 for the female participants (eFig. 4). Male SMG lower than 1713 and female SMG lower than 1318 were defined as the L-SMG group, with male SMG higher than 1713 and female SMG higher than 1318 defined as the H-SMG group. Compared with the H-SMG group, the L-SMG group was older (age ≥ 60 years: 70.8% vs. 54.0%; *p* = 0.001), had more male patients (83.1% vs. 68.8%; *p* = 0.001), and had larger tumors (tumor size ≥4 cm: 63.4% vs 50.4%; *p* < 0.001) (eTable 7).

In terms of short-term outcome, the incidence of postoperative complications in the L-SMG group was higher (27.6% vs. 9.3%; *p* = 0.001). The incidence of major complications also was higher in the L-SMG group than in the H-SMG group (11.1% vs. 2.1%; *p* < 0.001). The proportion of patients with delayed chemotherapy is highe than that of normal chemotherapy (29.2% vs. 19.8%; *p* = 0.005; eFig. 5). Compared with the H-SMG group, the L-SMG group had significantly worse 3-year OS (51% vs 89%, *p* < 0.001), DFS (41% vs 86%; *p* < 0.001), and RFS (41% vs. 85%, *p* < 0.001).

**Fig. 3:**
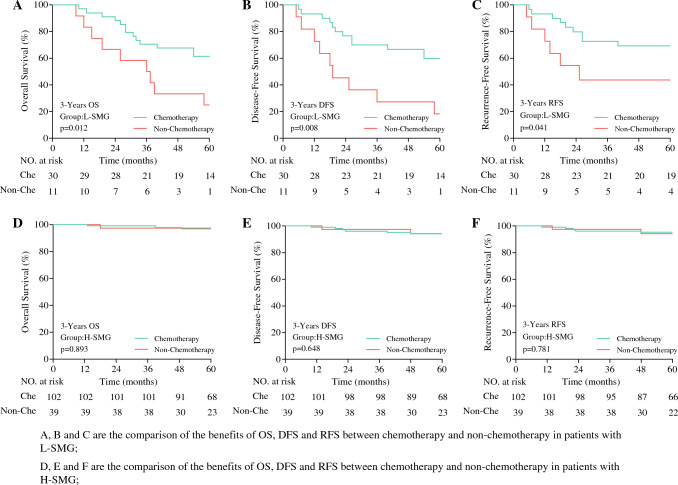
Comparison of chemotherapy benefits between high- and low-SMG groups of patients with postoperative pathological stage II. SMG, skeletal muscle gauge

**Fig. 4:**
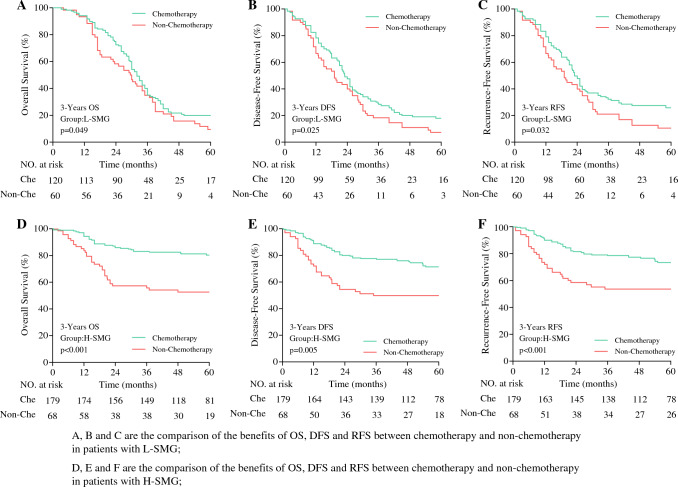
Comparison of chemotherapy benefits between high- and low-SMG groups in patients with postoperative pathological stage III. SMG, skeletal muscle gauge

Further analysis of the benefits of chemotherapy showed that among the patients with stage II disease, the L-SMG patients in the chemotherapy group had a significantly better 3-year OS (71% vs. 58%; *p* = 0.012), DFS (71% vs. 42%; *p* = 0.008), and RFS (73% vs 42%; *p* = 0.041) than those in the non-chemotherapy group. However, the H-SMG group showed no significant difference in OS (96% vs 95%; *p* = 0.893), DFS (97% vs 95%; *p* = 0.648), or RFS (97% vs 96%; *p* = 0.781) (Fig. 3). For the patients with stage III disease, regardless of low or high SMG, those who received chemotherapy had better 3-year OS, DFS, and RFS than those without chemotherapy (OS: L-SMG group [45% vs 35%; *p* = 0.049], H-SMG group [81% vs 61%; *p* < 0.001]; DFS: L-SMG group [35% vs 15%; *p* = 0.025], H-SMG group [78% vs 54%; *p* = 0.005]; RFS [L-SMG group [36% vs 16%; *p* = 0.032], H-SMG group [79% vs 53%; *p* < 0.001]; Fig. 4).

## Discussion

For the first time, we conducted a prospective study using a large sample of resectable gastric cancer patients to assess the potential value of SMG in both short- and long-term treatment outcomes. This study aimed to complement the existing definition of sarcopenia and provide timely clinical care for patients with sarcopenia.

The findings showed that SMG is a robust indicator for predicting both short- and long-term outcomes for patients with gastric cancer. Furthermore, SMG outperformed individual muscle quantity (SMI) and muscle quality (SMRA) in predicting postoperative complications and long-term results. In conclusion, SMG, as a unique muscle parameter, has greater predictive value than SMI and SMRA alone. It can effectively guide clinical treatment decisions for patients with locally advanced gastric cancer.

Previous retrospective studies, including our study, have provided initial evidence suggesting a correlation between sarcopenia and poor prognosis in gastric cancer.^26,27^ In the 2019 consensus, the Asian Working Group for sarcopenia defined sarcopenia as “age-related muscle loss and physical dysfunction.”^28^ Frequently, CT is used to assess muscle quantity and quality and often is included in the preoperative examination of tumor patients. However, there are discrepancies in evaluating sarcopenia based on CT regarding muscle quantity and quality, and the threshold values differ based on age, sex, and race.^29^ As a result, different studies may yield entirely distinct outcomes. Tegels et al.^30^ found that sarcopenia is very common among patients with gastric cancer (57.7%). However, their study did not observe a significant impact of sarcopenia on postoperative complications or mortality rates.^30^ The study by Tegels et al.^30^ defined sarcopenia based on SMI obtained from CT scanning. The findings indicated that sarcopenia, as defined by SMI, did not have a significant association with overall survival (OS) in pancreatic cancer. However, David et al.^31^ reported that whereas sarcopenia based on SMI did not affect OS, myosteatosis (characterized by low SMRA) was associated with a decline in the survival rate in pancreatic cancer.

The discrepancy in findings regarding the impact of sarcopenia on outcomes in gastric cancer and other cancers may arise from the use of different sarcopenia thresholds in various studies. Additionally, the results of the current study demonstrated that both SMI and SMRA could influence the short- and long-term outcomes for patients with gastric cancer to some degree. These findings imply that both muscle quantity and quality play a role in determining patient outcomes. The SMRA value reflects the inherent changes in the muscle structure of the body caused by tumor cachexia, which not only may be a simple decrease in the quantity of muscles but also may be related to the real mass changes of muscle tissue.^32^

Several recent studies have provided evidence that the SMG, which combines both SMI and SMRA, outperforms SMI and SMRA alone in predicting adverse outcomes in patients. This suggests that SMG may provide a more comprehensive and accurate assessment of muscle quantity and quality, leading to improved prognostic accuracy for patients.^10,33^ Our results also showed that SMG > SMI > SMRA > SMI in predicting postoperative complications, whereas SMG > SMI > SMRA in predicting long-term outcomes. In different outcomes, SMI and SMRA show different predictive performances, indicating that they have their advantages and complement each other in predicting outcomes. In addition, previous studies have shown that skeletal muscle mass begins to deteriorate significantly after age older than 50 years, and that muscle loss increases linearly with age.^34^ We also found more patients older than 60 years in the L-SMG group (L-SMG [70.8%] vs H-SMG [54.0%]). Furthermore, we analyzed the correlation between age and body composition parameters and found that BMI and SMI were not correlated with age, whereas SMG was negatively correlated with age. This shows that SMG combines not only the quantity and quality of skeletal muscle but also the trend of the age, which is a more effective prediction index in theory.^10^

Sarcopenia, caused by the cumulative decline of multiple physiologic systems, is a sign of weakness in patients with cancer. It is characterized by impaired function, reduced reserves, poor stress resistance, and easy-to-produce adverse effects.^35^ Previous studies have indeed demonstrated that abnormal fat accumulation within skeletal muscle plays a significant role in energy metabolism disorders, including conditions such as type 2 diabetes and insulin resistance.^36^ These metabolic disorders may lead to decreased tolerance to surgical trauma and stress. This explains the high incidence of postoperative complications in patients with low SMG levels to some extent. However, the mechanisms of sarcopenia and increased toxicity of chemotherapy are not clear. Some researchers believe that this is related to the pharmacokinetic distribution of chemotherapeutic drugs.^37^

Shachar et al.^38^ also found that the incidence of toxicity-related complications increased significantly among patients who have metastatic breast cancer with low SMG when receiving taxane chemotherapy and that drug doses usually are calculated based on body surface area (BSA). However, patients with similar BSA and BMI may have significantly different body compositions. This calculation method may be misleading because it does not consider body composition or lean body weight, which can lead to an overdose of antineoplastic drugs per unit of body weight and may have serious side effects.^39^ In this study, we found that patients with low SMG were prone to delayed chemotherapy, which may be one of the reasons for poor long-term outcomes among patients with low SMG.

Postoperative adjuvant chemotherapy has become the standard treatment for patients with locally advanced gastric cancer.^40^ However, in clinical practice, patients often have poor compliance with chemotherapy due to weight loss and eventually fail to complete the established chemotherapy cycle.^41^ Our results show that adjuvant chemotherapy can effectively prolong the survival of patients with low SMG (regardless of stage II or III disease). The results further show that the body composition index should be used to evaluate the patient’s physical condition rather than his or her weight alone. In addition, we did not find any benefit from adjuvant chemotherapy for patients with stage II and H-SMG, which may be related to the better survival status of patients with stage II disease (3-year OS for chemotherapy [96%] vs non-chemotherapy [95%]).

This study had some limitations. First, although this pooled analysis study provides more real-world data for evaluation of SMG, the three independent clinical studies related to laparoscopic gastric surgery have their research focuses. For example, some patients in the FUGES-001 study were in the early stage of the disease, whereas patients in the FUGES-02 and CLASS-04 studies were mainly in the advanced stage of disease and excluded all neoadjuvant patients. Second, because this study was a prospective pooled analysis, it did not evaluate the patient’s physical performance and muscle strength measurement (e.g., walking speed and grip strength measurement). Third, the patients in this study all were from China, and the applicability of the findings needs to be verified by people in the West and different regions. Nevertheless, this study collected data from three independent prospective trials with similar inclusion criteria, treatment methods, and postoperative management modes, and analyzed the applicability and effectiveness of SMG to include the largest number of patients, which greatly enhanced the evidence level of evidence-based medicine in this study.

## Conclusion

This study aimed to develop and verify the potential predictive value of a new muscle mass parameter for resectable gastric cancer. The study of muscle parameters found that SMI and SMRA provide independent and complementary information through prospective large-sample data, and that the product of these two indicators, the SMG, can effectively predict short- and long-term outcomes. As a new muscle parameter index, SMG is worthy of further study.

### Supplementary Information

Below is the link to the electronic supplementary material.

Supplementary file1 (DOCX 1077 KB)
