# Correction: Predictive Value of a New Muscle Parameter in Patients with Resectable Gastric Cancer: A Pooled Analysis of Three Prospective Trials

**DOI:** 10.1245/s10434-024-15069-3

**Published:** 2024-02-15

**Authors:** Qing Zhong, Jiao-Bao Huang, Jun Lu, Li-Wei Xue, Guang-Tan Lin, Jian-Wei Xie, Jian-Xian Lin, Chao-Hui Zheng, Chang-Ming Huang, Ping Li

**Affiliations:** 1 Department of Gastric Surgery, Fujian Medical University Union Hospital, Fuzhou, Fujian China; 2 Department of Gastrointestinal Surgery, The Second Affiliated Hospital of Nanchang University, Nanchang, China; 3 Department of Radiology, Fujian Medical University Union Hospital, Fuzhou, China

**Correction to: Ann Surg Oncol** 10.1245/s10434-024-14913-w

In the original online version of this article the Upper and Lower column headings in Tables 2 and 3 were transposed. The original article was corrected.

