# Supplementary material for: Predictive Value of a New Muscle Parameter in Patients with Resectable Gastric Cancer: A Pooled Analysis of Three Prospective Trials
Source: Ann Surg Oncol. 2024 Jan 25;31(5):3005–16. doi: 10.1245/s10434-024-14913-w (PMC10997550; doi:10.1245/s10434-024-14913-w)

**Supplementary table and figures**

**eTable 1** Eligibility Criteria in the FUGES-001 Study

**eTable 2** Eligibility Criteria in the FUGES-002 Study

**eTable 3** Eligibility Criteria in the CLASS-04 Study

**eTable 4** Classification of postoperative complications

**eTable 5** Univariate analysis of the relationship between muscle parameters and postoperative complications

**eTable 6** Univariate analysis of overall survival,disease-free survival and recurrence-free survival of all patients

**eTable 7** Comparison of clinical baseline data between L-SMG and H-SMG

**eFigure 1** Representative skeletal muscle area and skeletal muscle radiation attenuation at the third lumber vertebra

**eFigure 2** Correlation analysis between skeletal muscle parameters and baseline data

**eFigure 3** ROC and DCA curves compare the predictive performance of different muscle parameters for postoperative complications

**eFigure 4** Interception of SMG optimal cut-off point value

**eFigure 5** Comparison of short-outcome between L-SMG and H-SMG

**eTable 1** Eligibility Criteria in the FUGES-001 Study

| **Inclusion Criteria** |
| --- |
| Age from over 19 to under 74 years |
| cT 1-4a(clinical stage tumor), N0-3, M0 at preoperative evaluation according to the American Joint Committee on Cancer (AJCC) Cancer Staging Manual Seventh Edition |
| Heart, lungs, kidneys and other vital organs function well, with no obvious surgical contraindications |
| Preoperative examination with no distant metastasis, no significantly enlarged lymph nodes around abdominal main artery, and tumor not a direct violation of the pancreas, spleen and other surrounding organs |
| American Society of Anesthesiology (ASA) score class I, II, or III |
| Written informed consent |
| **Exclusion Criteria** |
| Women during pregnancy or breast-feeding |
| Severe mental disorder |
| History of previous upper abdominal surgery (except laparoscopic cholecystectomy) |
| Enlarged splenic hilar lymph nodes with integration into a mass and surrounding the blood vessels |
| History of unstable angina or myocardial infarction within past six months |
| History of cerebrovascular accident within past six months  including enlarged or bulky No.10 lymph nodes |
| History of continuous systematic administration of corticosteroids within one month |
| History of previous neoadjuvant chemotherapy or radiotherapy |
| T4b tumors |
| Emergency surgery due to complication (bleeding, obstruction or perforation) caused by gastric cancer |
| FEV1(Forced expiratory volume in one second)＜50% of predicted values |

**eTable 2** Eligibility Criteria in the FUGES-002 Study

| **Inclusion Criteria** |
| --- |
| Age between 18 and 75 years |
| Endoscopic biopsy confirmed primary gastric adenocarcinoma of the middle or upper third stomach not invading the greater curvature |
| cT2-4a, N-/+, M0 at preoperative evaluation according to the AJCC Cancer Staging Manual, 7th Edition |
| No distant metastasis, no significantly enlarged lymph nodes around abdominal main artery, no direct invasion of pancreas, spleen or other adjacent organs in the preoperative examinations |
| Performance status of 0 or 1 on ECOG scale |
| ASA class I, II, or III |
| Written informed consent |
| **Exclusion Criteria** |
| Women during breast-feeding or pregnancy |
| Severe mental disorder |
| Previous upper abdominal surgery (except laparoscopic cholecystectomy) |
| Previous gastrectomy, endoscopic mucosal resection, or endoscopic submucosal dissection |
| Other malignant disease within the past 5 years |
| Enlarged or bulky regional lymph node (diameter over 3cm) supported by preoperative imaging including enlarged or bulky No.10 lymph nodes |
| Previous neoadjuvant chemotherapy or radiotherapy |
| Unstable myocardial infarction, angina, or cerebrovascular accident within the past 6 months |
| History of continuous systematic administration of corticosteroids within one month |
| FEV1＜50% of predicted values |
| Requirement of simultaneous surgery for other disease |
| Emergency surgery due to complication (bleeding, obstruction or perforation) caused by gastric cancer |

**Abbreviations**: **AJCC**: American Joint Committee on Cancer; **ASA**: American Society of Anesthesiology; **ECOG**: Eastern Cooperative Oncology Group; **FEV1**: forced expiratory volume in 1 second.

**eTable 3** Eligibility Criteria in the CLASS-04 Study

| **Inclusion Criteria** |
| --- |
| Age between 18 and 75 years |
| Endoscopic biopsy confirmed primary gastric adenocarcinoma of the middle or upper third stomach not invading the greater curvature |
| cT2-4a, N-/+, M0 at preoperative evaluation according to the AJCC Cancer Staging Manual, 7th Edition |
| No distant metastasis, no significantly enlarged lymph nodes around abdominal main artery, no direct invasion of pancreas, spleen or other adjacent organs in the preoperative examinations |
| Performance status of 0 or 1 on ECOG scale |
| ASA class I, II, or III |
| Written informed consent |
| **Exclusion Criteria** |
| Women during breast-feeding or pregnancy |
| Severe mental disorder |
| Previous upper abdominal surgery (except laparoscopic cholecystectomy) |
| Previous gastrectomy, endoscopic mucosal resection, or endoscopic submucosal dissection |
| Other malignant disease within the past 5 years |
| Enlarged or bulky regional lymph node (diameter over 3cm) supported by preoperative imaging including enlarged or bulky No.10 lymph nodes |
| Previous neoadjuvant chemotherapy or radiotherapy |
| Unstable myocardial infarction, angina, or cerebrovascular accident within the past 6 months |
| History of continuous systematic administration of corticosteroids within one month |
| FEV1＜50% of predicted values |
| Requirement of simultaneous surgery for other disease |
| Emergency surgery due to complication (bleeding, obstruction or perforation) caused by gastric cancer |
| Patients who need combined splenectomy because of obvious tumor infiltration in spleen and splenic vessels |

**Abbreviations**: **AJCC**: American Joint Committee on Cancer; **ASA**: American Society of Anesthesiology; **ECOG**: Eastern Cooperative Oncology Group; **FEV1**: forced expiratory volume in 1 second.

**eTable 4** Classification of postoperative complications

| **Postoperative Complications** | **N (%)** |
| --- | --- |
| Intraabdominal bleeding | 5(0.6) |
| Anastomotic leak | 15(2.0) |
| Intestinal obstruction | 4(0.5) |
| Wound infection | 4(0.5) |
| Abdominal infection | 23(3.2) |
| Pneumonia | 71(9.9) |
| Single organ dysfunction(Cardio-cerebral vascular system) | 4(0.5) |
| Single organ dysfunction(Abnormal liver function) | 9(1.2) |
| Abdominal lymphatic leakage | 12(2.0) |
| **Major Postoperative Complications (CD ≥III)** |  |
| Intraabdominal bleeding | 1(0.13) |
| Anastomotic leak | 3(0.42) |
| Intestinal obstruction | 1(0.13) |
| Wound infection | 2(0.28) |
| Abdominal infection | 13(1.8) |
| Pneumonia | 26(3.6) |
| Single organ dysfunction(Cardio-cerebral vascular system) | 1(0.13) |
| Abdominal lymphatic leakage | 7(1.0) |
| **CD** indicates Clavien-Dindo classification. | |

**eTable 5** Univariate analysis of the relationship between muscle parameters and postoperative complications

|  | **Postoperative Complications** | | | |
| --- | --- | --- | --- | --- |
|  |  | 95%CI | |  |
|  | OR | Upper | Lower | *P* |
| Age（year） |  |  |  |  |
| < 60 | Ref. |  |  |  |
| ≥ 60 | 1.71 | 1.13 | 2.53 | **0.016** |
| Gender |  |  |  |  |
| Male | Ref. |  |  |  |
| Female | 0.98 (0.57-1.69) | 0.57 | 1.69 | 0.939 |
| ECOG |  |  |  |  |
| 0 | Ref. |  |  |  |
| ≥ 1 | 1.62 (1.03-2.61) | 1.07 | 2.54 | **0.033** |
| Tumor size（cm） |  |  |  |  |
| < 4 | Ref. |  |  |  |
| ≥ 4 | 1.37 (0.90-2.07) | 0.90 | 2.07 | 0.139 |
| Intraoperative bleeding volume | 1.00 (0.99-1.02) | 0.99 | 1.02 | 0.514 |
| Operation time | 1.01 (0.99-1.01) | 0.99 | 1.01 | 0.091 |
| pTNM stage |  |  |  |  |
| I | Ref. |  |  |  |
| II | 0.95 (0.42-2.16) | 0.42 | 2.16 | 0.909 |
| III | 0.88 (0.42-2.16) | 0.46 | 2.23 | 0.732 |
| BMI（kg/m^2^） |  |  |  |  |
| < 25 | Ref. |  |  |  |
| ≥ 25 | 2.56 (1.65-4.87) | 1.81 | 4.68 | **0.032** |
| SMI | 0.95 (0.92-0.96) | 0.91 | 0.96 | **0.001** |
| SMRA | 0.86 (0.84-0.90) | 0.83 | 0.90 | **0.001** |
| SMG | 0.99 (0.98-0.99) | 0.98 | 0.99 | **0.001** |
| **ECOG:** Eastern cooperative oncology group; **BMI**: Body mass index; **SMI**: Skeletal muscle index, **SMRA:** Skeletal muscle radiation attenuation, **SMG:** Skeletal muscle gauge; | | | | |

**eTable 6** Univariate analysis of overall survival,disease-free survival and recurrence-free survival of all patients

|  | **Overall Survival** | | | |  | **Disease-Free Survival** | | | |  | **Recurrence-Free Survival** | | | |
| --- | --- | --- | --- | --- | --- | --- | --- | --- | --- | --- | --- | --- | --- | --- |
|  | (95%CI) | | | |  | (95%CI) | | | |  | (95%CI) | | | |
|  | HR | Upper | Lower | *P* |  | HR (95%CI) | Upper | Lower | *P* |  | HR (95%CI) | Upper | Lower | *P* |
| Age (year) |  |  |  |  |  |  |  |  |  |  |  |  |  |  |
| < 60 | Ref. |  |  |  |  | Ref. |  |  |  |  | Ref. |  |  |  |
| ≥ 60 | 0.86 | 0.49 | 1.51 | 0.597 |  | 1.08 | 0.85 | 1.34 | 0.555 |  | 1.22 | 0.68 | 2.16 | 0.507 |
| Gender |  |  |  |  |  |  |  |  |  |  |  |  |  |  |
| Male | Ref. |  |  |  |  | Ref. |  |  |  |  | Ref. |  |  |  |
| Female | 0.97 | 0.73 | 1.29 | 0.833 |  | 1.09 | 0.84 | 1.42 | 0.523 |  | 0.60 | 0.31 | 1.17 | 0.136 |
| ECOG |  |  |  |  |  |  |  |  |  |  |  |  |  |  |
| 0 | Ref. |  |  |  |  | Ref. |  |  |  |  | Ref. |  |  |  |
| ≥1 | 1.22 | 0.66 | 2.27 | 0.522 |  | 0.87 | 0.69 | 1.11 | 0.264 |  | 0.74 | 0.42 | 1.30 | 0.294 |
| Tumor size (cm) |  |  |  |  |  |  |  |  |  |  |  |  |  |  |
| < 4 | Ref. |  |  |  |  | Ref. |  |  |  |  | Ref. |  |  |  |
| ≥ 4 | 2.43 | 1.85 | 3.20 | **0.001** |  | 2.23 | 1.76 | 2.93 | **0.001** |  | 4.61 | 2.29 | 9.24 | **0.001** |
| Postoperative complication |  |  |  |  |  |  |  |  |  |  |  |  |  |  |
| Yes | Ref. |  |  |  |  | Ref. |  |  |  |  | Ref. |  |  |  |
| No | 1.18 | 0.58 | 2.37 | 0.652 |  | 0.88 | 0.63 | 1.23 | 0.877 |  | 1.14 | 0.58 | 2.23 | 0.700 |
| pTNM stage |  |  |  |  |  |  |  |  |  |  |  |  |  |  |
| I | Ref. |  |  |  |  | Ref. |  |  |  |  | Ref. |  |  |  |
| II | 3.39 | 1.31 | 8.81 | **0.012** |  | 4.20 | 1.64 | 10.75 | **0.003** |  | 3.80 | 2.46 | 9.55 | **0.005** |
| III | 15.92 | 6.57 | 38.71 | **0.001** |  | 18.01 | 7.43 | 43.69 | **0.001** |  | 16.32 | 8.64 | 40.33 | **0.001** |
| BMI (kg/m^2^) |  |  |  |  |  |  |  |  |  |  |  |  |  |  |
| < 25 | Ref. |  |  |  |  | Ref. |  |  |  |  | Ref. |  |  |  |
| ≥ 25 | 1.64 | 0.84 | 3.21 | 0.146 |  | 0.83 | 0.61 | 1.14 | 0.250 |  | 1.18 | 0.63 | 2.23 | 0.608 |
| Adjuvant chemotherapy |  |  |  |  |  |  |  |  |  |  |  |  |  |  |
| Yes | Ref. |  |  |  |  | Ref. |  |  |  |  | Ref. |  |  |  |
| No | 1.43 | 1.11 | 1.84 | **0.005** |  | 1.28 | 1.01 | 1.63 | **0.043** |  | 1.25 | 1.16 | 1.72 | **0.008** |
| SMI | 0.93 | 0.89 | 0.96 | **0.001** |  | 0.92 | 0.91 | 0.93 | **0.001** |  | 0.93 | 0.91 | 0.94 | **0.001** |
| SMRA | 0.93 | 0.89 | 0.97 | **0.001** |  | 0.96 | 0.95 | 0.97 | **0.001** |  | 0.96 | 0.95 | 0.98 | **0.001** |
| SMG | 0.99 | 0.97 | 0.99 | **0.001** |  | 0.99 | 0.97 | 0.99 | **0.001** |  | 0.99 | 0.99 | 0.99 | **0.001** |
| **ECOG:** Eastern cooperative oncology group; **BMI**:Body mass index; **SMI**:Skeletal muscle index, **SMRA:**Skeletal muscle radiation attenuation, **SMG:**Skeletal muscle gauge; | | | | | | | | | | | | | | |

**eTable 7** Comparison of clinical baseline data between L-SMG and H-SMG

| **Characteristics** | **L-SMG**  **N=243** | 1. **SMG**   **N=474** | ***P*** |
| --- | --- | --- | --- |
| Age, No. (%) |  |  | **0.001** |
| < 60 | 71（29.2） | 218（46.0） |  |
| ≥ 60 | 172（70.8） | 256（54.0） |  |
| Gender, No. (%) |  |  | **0.001** |
| Male | 202（83.1） | 326（68.8） |  |
| Female | 41（16.9） | 148（31.2） |  |
| ECOG, No. (%) |  |  | 0.131 |
| 0 | 127（52.3） | 277（58.4） |  |
| ≥ 1 | 116（47.7） | 197（41.6） |  |
| BMI, No. (%) |  |  | 0.689 |
| < 25 | 199（81.9） | 382（80.6） |  |
| ≥ 25 | 44（18.1） | 92（19.4） |  |
| Tumor location, (%) |  |  | 0.062 |
| Upper | 155（63.8） | 288（60.8） |  |
| Middle | 55（22.6） | 141（29.7） |  |
| Lower | 33（13.6） | 45（9.5） |  |
| Tumor size, No. (%) |  |  | **0.001** |
| < 4 | 89（36.6） | 235（49.6） |  |
| ≥ 4 | 154（63.4） | 239（50.4） |  |
| Intraoperative bleeding volume, (ml) | 65±58 | 67±55 | 0.471 |
| Operation time, (min) | 180±38 | 185±40 | 0.201 |
| pTNM stage, No. (%) |  |  | **0.001** |
| I | 22（9.1） | 86（18.1） |  |
| II | 41（16.9） | 141（29.7） |  |
| III | 180（74.1） | 247（52.1） |  |
| Postoperative complication, , No. (%) |  |  | **0.001** |
| Yes | 67（27.6） | 44（9.3） |  |
| No | 176（72.4） | 430（90.7） |  |
| Delayed  chemotherapy , No. (%) |  |  | **0.003** |
| Yes | 71（29.2） | 94（19.8） |  |
| No | 172（70.8） | 380（80.2） |  |
| **ECOG:** Eastern cooperative oncology group; **BMI**: Body mass index; **SMI**: Skeletal muscle index, **SMRA:** Skeletal muscle radiation attenuation, **SMG:** Skeletal muscle gauge | | | |

**eFigure 1** Representative skeletal muscle area and skeletal muscle radiation attenuation at the third lumber vertebra


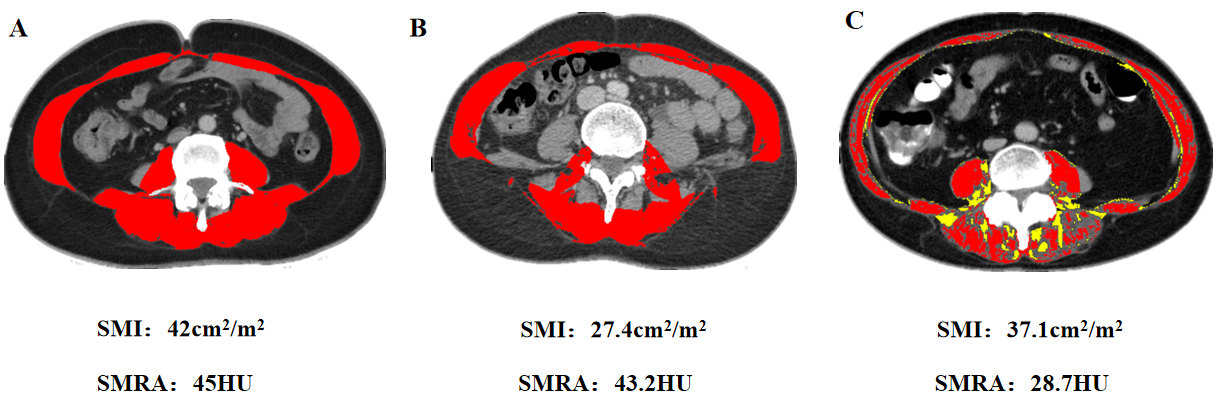


**Red**:Skeletal muscle area(SMA);**Yellow**:Muscle infiltrated with fat;(**A**)Normal skeletal muscle;(**B**)Low skeletal muscle index(L-SMI);(**C**)Low skeletal muscle radiation attenuation(L-SMRA);

**eFigure 2** Correlation analysis between skeletal muscle parameters and baseline data


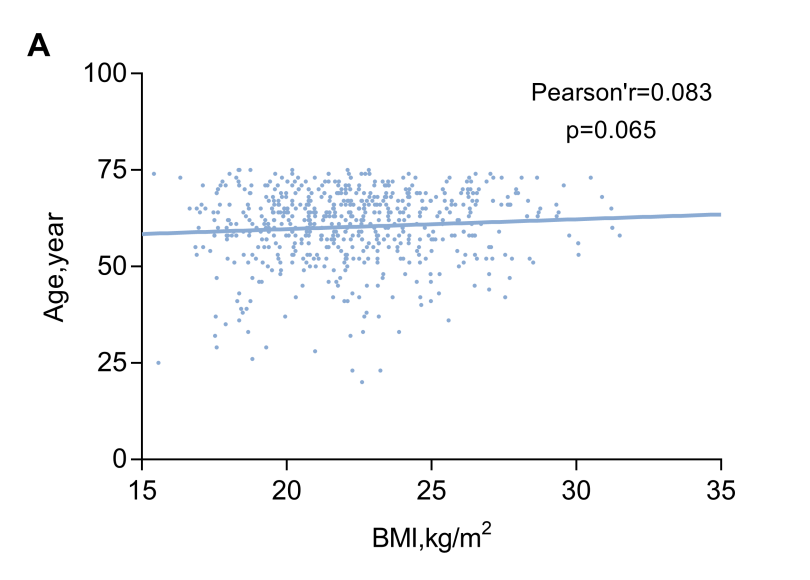

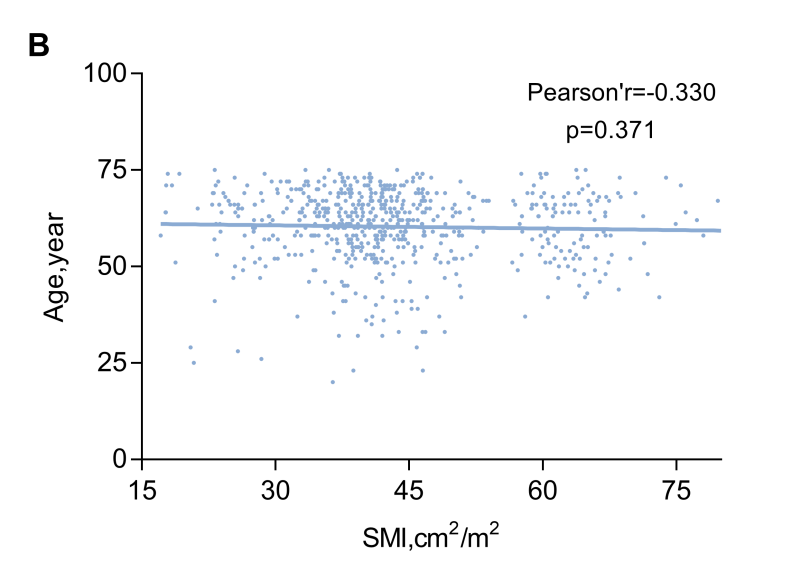

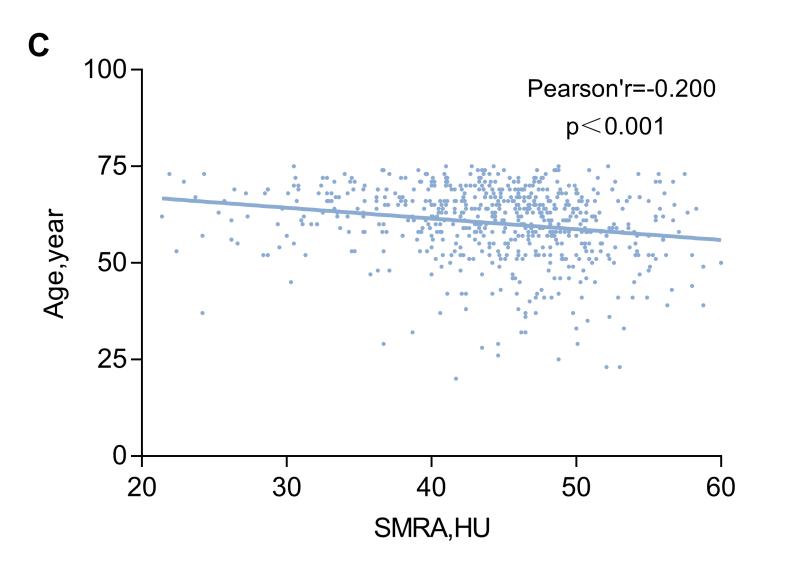

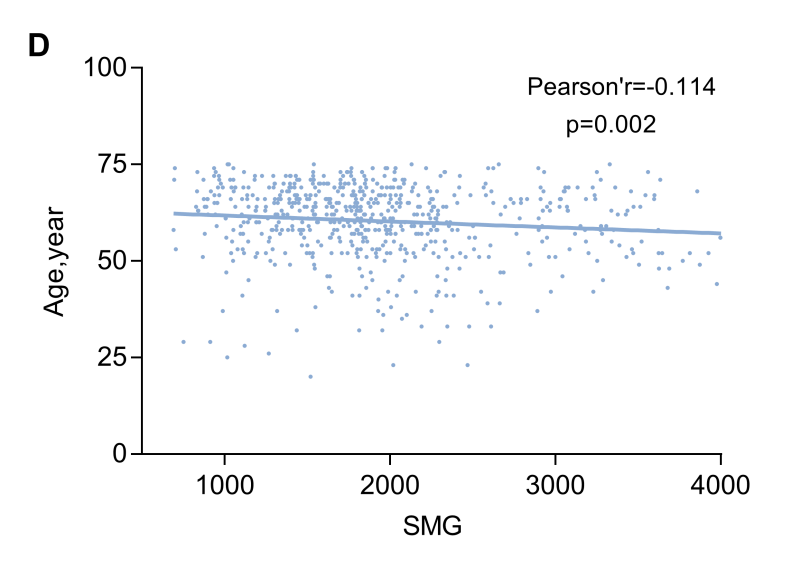

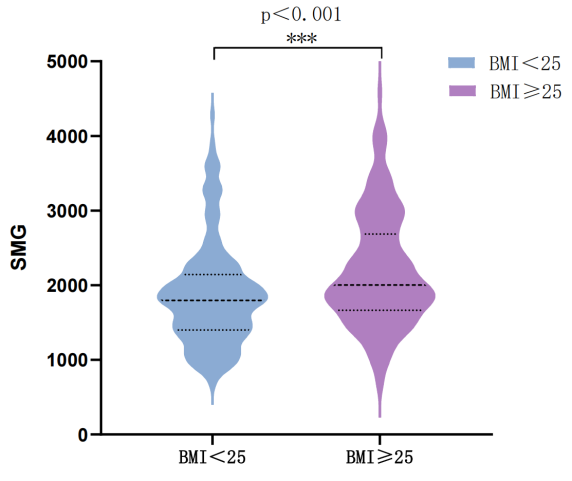

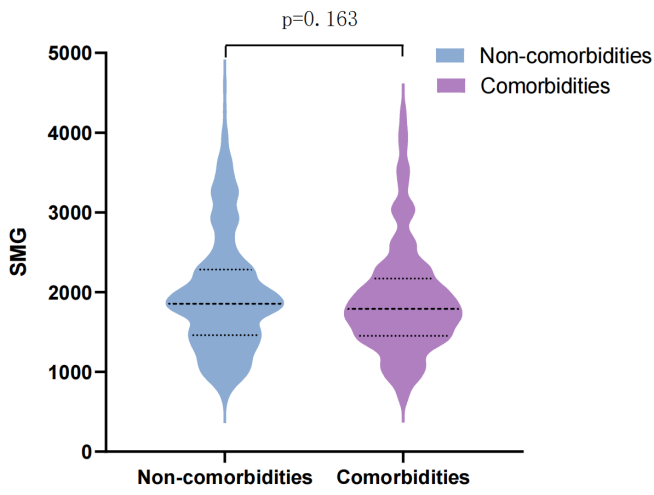

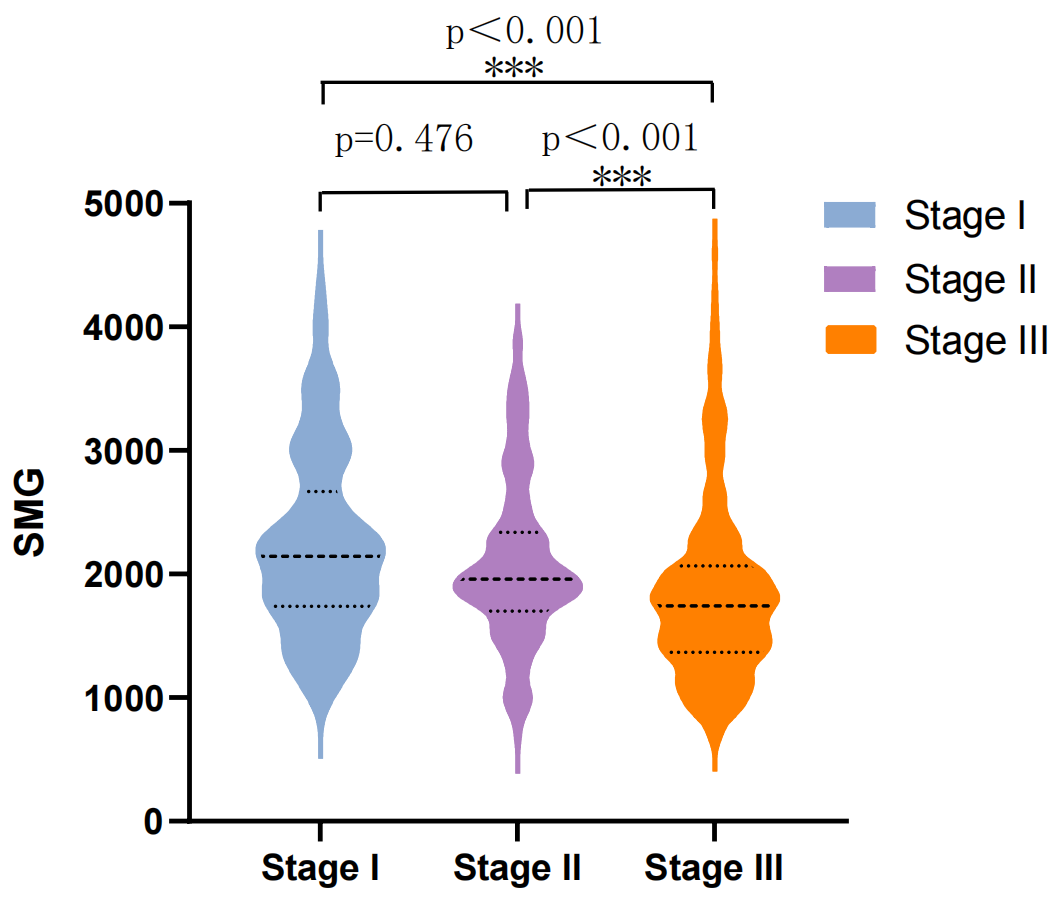


**A**:BMI has no correlation with age; **B**:SMI has no correlation with age;

**C**:SMRA is negatively correlated with age; **D**:SMG is negatively correlated with age;

**E**:BMI is positively correlated with SMG； **F**:comorbidity has no correlation with SMG;

**G**:Pathological staging was negatively correlated with SMG.

**eFigure 3** ROC and DCA curves compare the predictive performance of different muscle parameters for postoperative complications


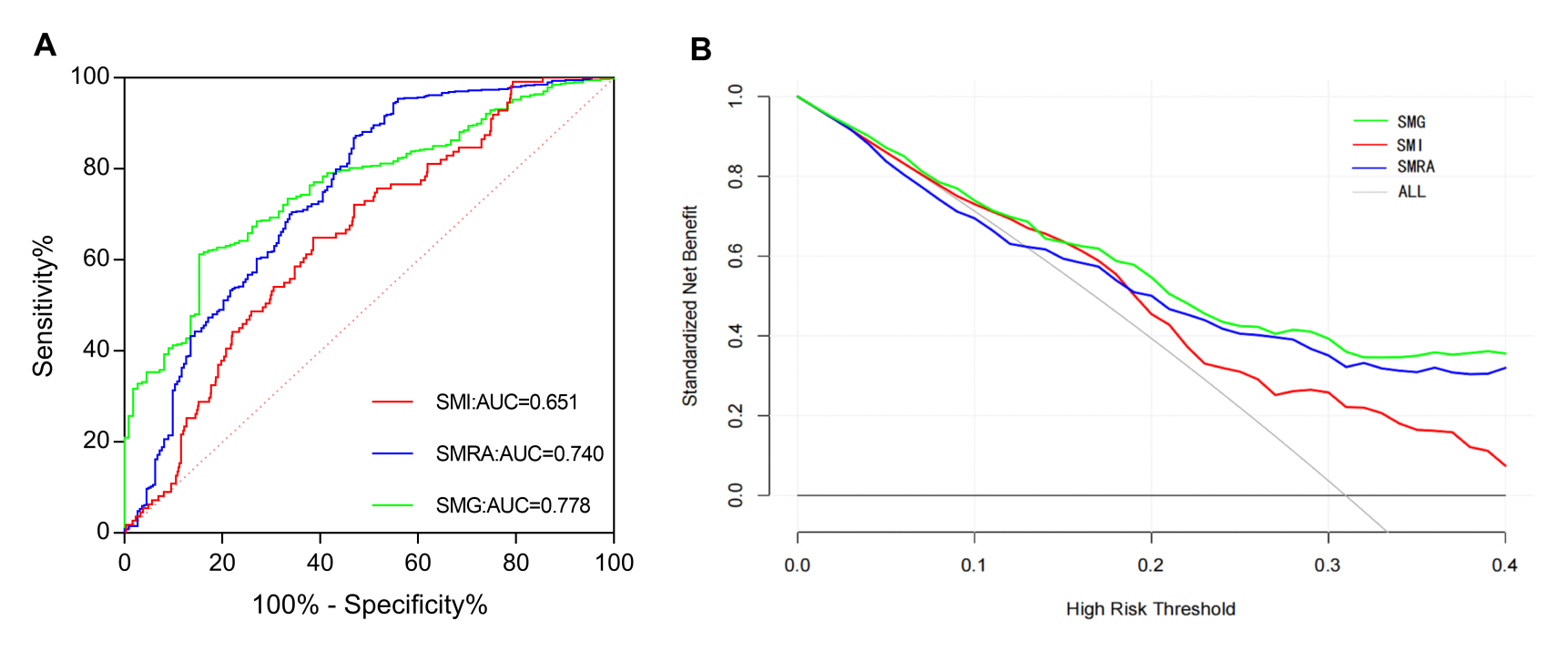


**A**: ROC curve compares the predictive performance of three groups of muscle parameters;

**B**: DCA curve compares the predictive performance of three groups of muscle parameters;

**eFigure 4** Interception of SMG optimal cut-off point value

**A**


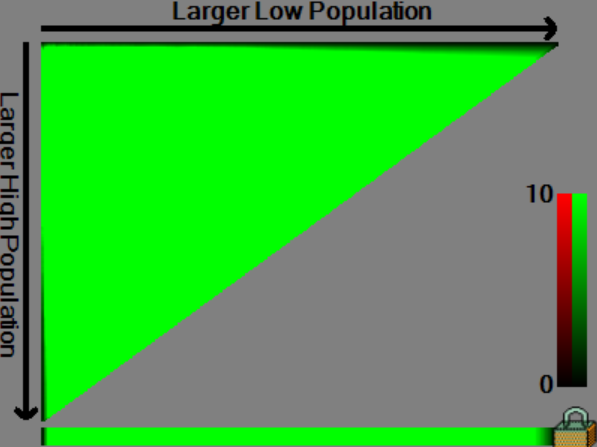

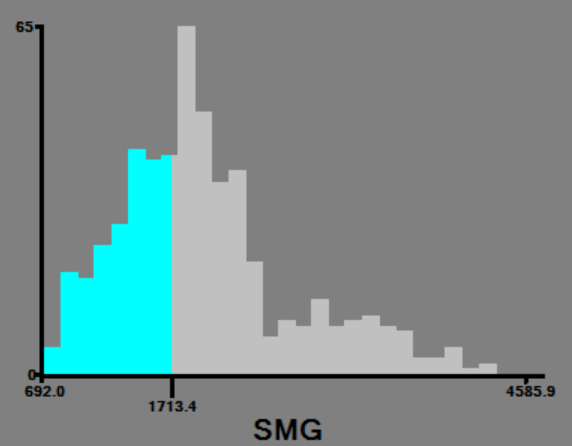

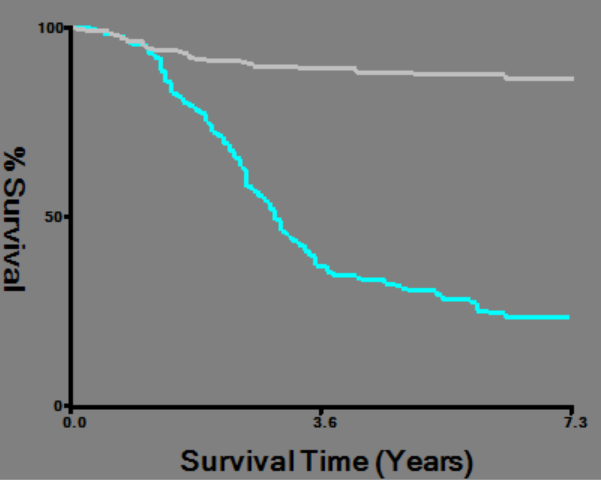


**B**


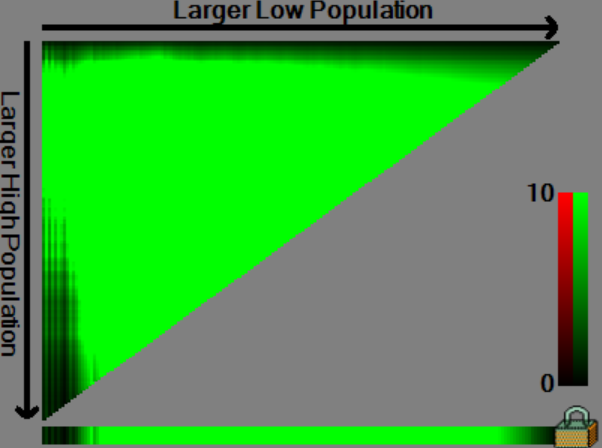

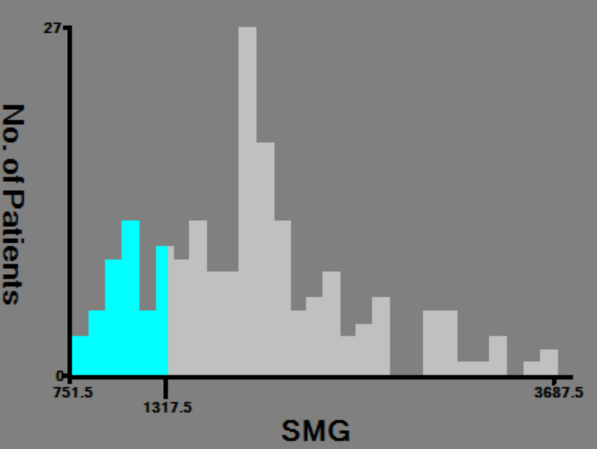

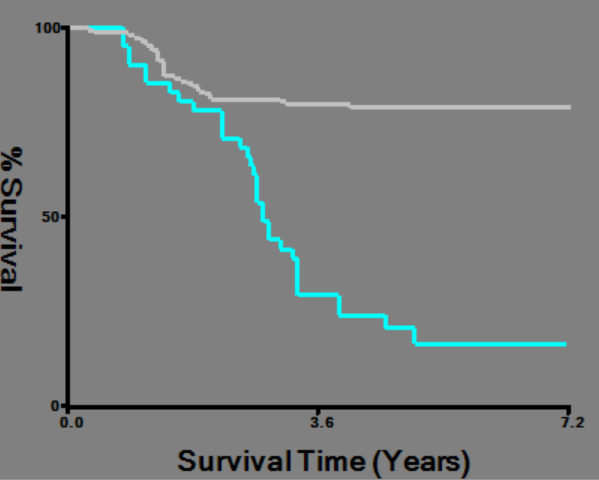


**A** is a male patient; **B** is a female patient;

**eFigure 5** Comparison of short-outcome between L-SMG and H-SMG


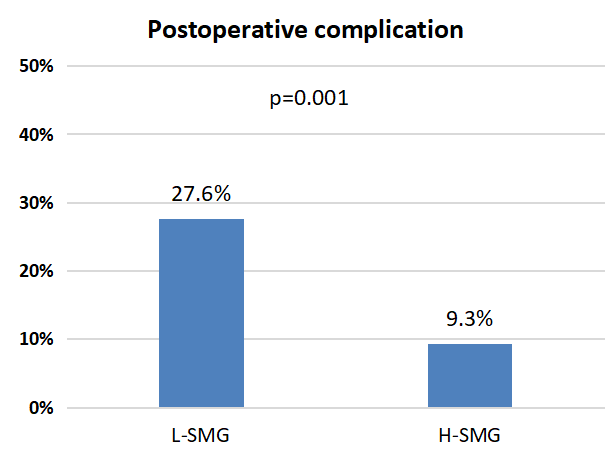

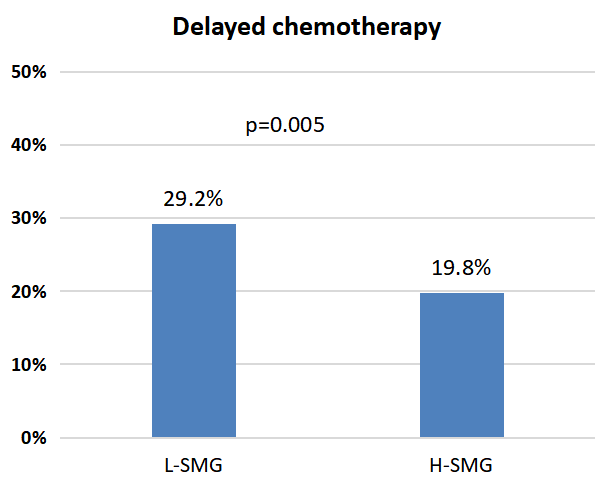


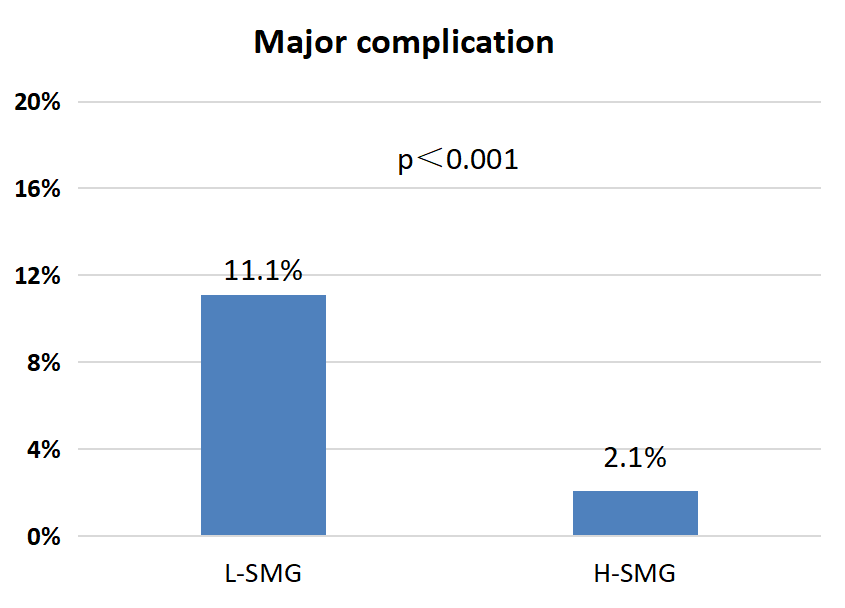

Supplement: Supplementary file 1 — Supplementary file1 (DOCX 1077 KB) [file 10434_2024_14913_MOESM1_ESM.docx]
